# Clinical efficacy and safety of abatacept in methotrexate-naive patients with early rheumatoid arthritis and poor prognostic factors

**DOI:** 10.1136/ard.2008.101121

**Published:** 2009-01-04

**Authors:** R Westhovens, M Robles, A C Ximenes, S Nayiager, J Wollenhaupt, P Durez, J Gomez-Reino, W Grassi, B Haraoui, W Shergy, S-H Park, H Genant, C Peterfy, J-C Becker, A Covucci, R Helfrick, J Bathon

**Affiliations:** 1 UZ Gasthuisberg, Leuven, Belgium; 2 Centro Médico Toluca, Metepec, México; 3 Hospital Geral de Goiânia, Goiânia, Brazil; 4 St Augustine’s Hospital, Durban, South Africa; 5 Klinikum Eilbek, Hamburg, Germany; 6 Cliniques Universitaires Saint-Luc, Université Catholique de Louvain, Brussels, Belgium; 7 Hospital Clinico Universidad De Santiago, A Coruna, Spain; 8 Clinica Reumatologica, Università Politecnica delle Marche, Ancona, Italy; 9 Institut de Rhumatologie de Montréal, Montreal, Quebec, Canada; 10 University of Alabama, Huntsville, Alabama, USA; 11 The Catholic University of Korea, Seoul, Korea; 12 University of California, San Francisco/Synarc, San Francisco, California, USA; 13 Synarc, Inc, San Francisco, California, USA; 14 Bristol-Myers Squibb, Princeton, New Jersey, USA; 15 John Hopkins University, Baltimore, Maryland, USA

## Abstract

**Objectives:**

To assess the efficacy and safety of abatacept in methotrexate-naive patients with early rheumatoid arthritis (RA) and poor prognostic factors.

**Methods:**

In this double-blind, phase IIIb study, patients with RA for 2 years or less were randomly assigned 1 : 1 to receive abatacept (∼10 mg/kg) plus methotrexate, or placebo plus methotrexate. Patients were methotrexate-naive and seropositive for rheumatoid factor (RF), anti-cyclic citrullinated protein (CCP) type 2 or both and had radiographic evidence of joint erosions. The co-primary endpoints were the proportion of patients achieving disease activity score in 28 joints (DAS28)-defined remission (C-reactive protein) and joint damage progression (Genant-modified Sharp total score; TS) at year 1. Safety was monitored throughout.

**Results:**

At baseline, patients had a mean DAS28 of 6.3, a mean TS of 7.1 and mean disease duration of 6.5 months; 96.5% and 89.0% of patients were RF or anti-CCP2 seropositive, respectively. At year 1, a significantly greater proportion of abatacept plus methotrexate-treated patients achieved remission (41.4% vs 23.3%; p<0.001) and there was significantly less radiographic progression (mean change in TS 0.63 vs 1.06; p = 0.040) versus methotrexate alone. Over 1 year, the frequency of adverse events (84.8% vs 83.4%), serious adverse events (7.8% vs 7.9%), serious infections (2.0% vs 2.0%), autoimmune disorders (2.3% vs 2.0%) and malignancies (0.4% vs 0%) was comparable for abatacept plus methotrexate versus methotrexate alone.

**Conclusions:**

In a methotrexate-naive population with early RA and poor prognostic factors, the combination of abatacept and methotrexate provided significantly better clinical and radiographic efficacy compared with methotrexate alone and had a comparable, favourable safety profile.

In rheumatoid arthritis (RA), persistent synovitis, early erosions and the presence of rheumatoid factor (RF) and anti-cyclic citrullinated peptide (CCP) type 2 antibodies are prognostic indicators of joint destruction and loss of function.^1^ ^2^ ^3^ The initiation of intensive treatment early in the course of disease is now an accepted paradigm in the treatment of RA, with an increasing emphasis on tight disease control and clinical remission as a treatment goal.^4^ ^5^

Studies comparing biological agents in combination with methotrexate compared with methotrexate alone have demonstrated significant benefit when treatment is initiated early.^6^ ^7^ ^8^ ^9^ ^10^ These trials have also highlighted that, although methotrexate monotherapy can be efficacious, it does not provide optimal disease control in a proportion of patients.

Abatacept is a soluble, fully human, recombinant fusion protein that selectively modulates the CD80/CD86:CD28 co-stimulatory signal for T-cell activation.^11^ The ability of abatacept to modulate the activation of T cells, including naive T cells, and the role of T cells in initiating disease^12^ suggests that abatacept has the potential to impact the progression of pathology early in the course of disease.

The sustained efficacy and safety of abatacept has previously been demonstrated in patients with moderate-to-severe established RA who have had an inadequate response to methotrexate^13^ and/or anti-tumour necrosis factor agents.^14^ Here, we report 1-year data from a study that assessed the efficacy, safety and tolerability of abatacept plus methotrexate compared with methotrexate alone, in methotrexate-naive patients with early RA (⩽2 years). The patients in this study represented a particularly poor prognosis population, because the inclusion criteria required all patients to have erosions and to be seropositive for RF and/or anti-CCP2, which are associated with poor radiological outcomes.^1^ ^2^ ^3^

## Patients and methods

### Patients

Eligible patients were 18 years of age or older, with RA^15^ for 2 years or less, at least 12 tender and 10 swollen joints, C-reactive protein (CRP) 0.45 mg/dl or greater, RF and/or anti-CCP2 seropositivity and radiographic evidence of bone erosion of the hands/wrists/feet. Patients were either methotrexate-naive or had previous exposure of 10 mg/week or less for 3 weeks or less, with none administered for 3 months before providing informed consent (there were no requirements relating to the reason for discontinuation of previous methotrexate therapy).

### Study design

This was a multi-national, randomised, double-blind, 2-year study (ClinicalTrials.gov identifier: NCT00122382). The protocol and patients’ informed consent received institutional review board/independent ethics committee approval, and the study was conducted in accordance with the Declaration of Helsinki and was consistent with International Conference on Harmonisation Good Clinical Practice. Patients, sites and the site conducting radiographic assessment remained blinded to treatment assignments until the end of the study.

Patients were randomly assigned 1 : 1 to receive abatacept (∼10 mg/kg according to weight range) plus methotrexate or placebo plus methotrexate for 1 year by intravenous infusion on days 1, 15 and 29, and every 4 weeks thereafter. Methotrexate was initially dosed at 7.5 mg/week and subsequently increased to 15 mg at week 4 and to 20 mg at week 8, at which dose it was maintained until study completion. Dose reduction was permitted to a minimum of 15 mg/week due to toxicity or intolerability.

Women who were pregnant or breastfeeding were excluded, and patients were required to practice effective contraceptive measures for the study duration. Patients were excluded if they had had active *Mycobacterium tuberculosis* (tuberculosis) requiring treatment within 3 years. Patients with a positive purified protein derivative test were eligible if treatment for latent tuberculosis had been initiated (according to local guidelines) and there was no evidence of active tuberculosis by chest *x* ray at enrollment.

Stable low-dose oral corticosteroids (⩽10 mg prednisone equivalent, daily) were permitted, plus up to two corticosteroid “pulses” (>10 mg prednisone or equivalent oral corticosteroids for at least three consecutive days or injectable corticosteroids) in any 6-month period. After 6 months, the addition of one non-biological disease-modifying antirheumatic drug (DMARD) was permitted.

### Clinical assessments

#### Efficacy assessments

##### Co-primary endpoints

Remission at year 1, defined as a disease activity score in 28 joints (DAS28; CRP) of less than 2.6;^16^ structural damage at year 1, measured using the Genant-modified Sharp scoring system^17^ ^18^ ^19^ total score (TS) with a maximum possible score of 290.

##### Secondary endpoints

These were assessed at year 1 and included American College of Rheumatology (ACR) 50 responses; major clinical response (MCR; ACR70 maintained for ⩾6 consecutive months); DAS28 (CRP) scores; Genant-modified Sharp erosion score (ES; maximum possible 145) and joint-space narrowing score (JSN; maximum possible 145); physical function (defined as an improvement of ⩾0.3 units from baseline in the health assessment questionnaire disability index; HAQ-DI)^20^ and health-related quality of life (HRQoL) measured using the short form (SF)-36, with an improvement of 3 units or more considered to be clinically meaningful.^21^

The proportion of patients achieving ACR70 and ACR90 responses and the proportion of patients without radiographic progression (change in TS from baseline of ⩽0) were also evaluated.

#### Safety assessments

All patients who received one dose of abatacept were evaluated, and adverse events and serious adverse events were classified using the Medical Dictionary for Regulatory Activities version 10.1. Autoimmune and infusional events were taken from a predefined list of events of interest. Acute infusional events were any adverse event occurring within 1 h after initiation of infusion.

### Statistical analysis

#### Power calculation

It was estimated that 500 patients randomly assigned 1 : 1 to each group would yield 99% power and detect a 20% difference in DAS28 (CRP)-defined remission rates at the 5% level (two-tailed test). A response rate of 15% at year 1 in the methotrexate group and an overall 15% dropout rate was assumed. Based on the hierarchical testing procedure for the co-primary endpoints, this sample size also allowed the detection of a treatment difference of 1.6 (common SD 5) with a power of 90% for mean change from baseline in TS.

#### Data analysis

Unless otherwise stated, efficacy analyses were performed on all patients randomly assigned and treated: for DAS28 (CRP)-defined remission, ACR and HAQ-DI responses; patients who discontinued were considered non-responders subsequent to discontinuation. For analysis of the co-primary endpoints, patients who received more than one corticosteroid pulse (oral >10 mg/day prednisone equivalent for ⩾3 consecutive days, intramuscularly or intravenously) during year 1, or who received more than two intra-articular injections (corticosteroid pulses counted towards the limit for intra-articular injections) from baseline to month 6 or month 6 to year 1 were classified as non-responders after that time. For mean change from baseline in DAS28 (CRP), HAQ-DI and SF-36, a last observation carried forward imputation was applied. For radiographic assessment, all available data were included. At year 1, missing data for TS, ES and JSN were imputed by linear extrapolation for patients with radiographs at baseline and either month 6 or discontinuation (or both). For the cumulative probability plot, the observed cumulative proportion (scores ranked from lowest to highest and presented as a cumulative proportion of all scores) was plotted against the actual change from baseline.

#### Sensitivity analysis

For the proportion of patients without radiographic progression, an analysis was performed in which all patients who received additional non-biological DMARD were considered non-responders. For DAS28 (CRP)-defined remission and the proportion of patients without radiographic progression, additional analyses were performed in which all patients who received additional non-biological DMARD and/or a steroid pulse were considered non-responders. For mean change from baseline to year 1 in TS, ES and JSN, an analysis was performed considering only completers.

#### Comparisons between treatment groups

For DAS28 (CRP)-defined remission, ACR, MCR and HAQ-DI responder rates at year 1, a continuity-corrected χ^2^ test was used. For change from baseline for DAS28 (CRP), HAQ-DI and SF-36, comparisons were based on an analysis of covariance model, including treatment as the main factor and baseline value as a covariate. A non-parametric analysis of covariance model was used for change from baseline to year 1 in TS, ES and JSN.

## Results

### Baseline demographics and characteristics, and patient disposition

**Figure 1:**
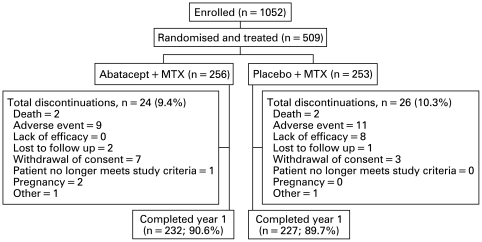
Patient disposition over 1 year. MTX, methotrexate.

Five hundred and nine patients (predominantly South American (40.3%) and European (36.0%)) were randomly assigned to receive abatacept plus methotrexate (n  =  256) or methotrexate alone (n  =  253). Demographics and baseline clinical characteristics were comparable between groups, with an overall mean disease duration of 6.5 months (table 1). At baseline, patients had a high disease activity evidenced by an overall mean DAS28 (CRP) score of 6.3, and mean tender and swollen joint counts of 31.0 and 22.4, respectively. In total, eight (3.1%) patients in the abatacept plus methotrexate group and two (0.8%) patients in the methotrexate alone group had received methotrexate before randomisation. The mean baseline TS was 7.1. A total of 491 (96.5%) and 453 (89.0%) patients was seropositive for RF and anti-CCP2, respectively; 86.1% of patients were seropositive for both. A total of 232 (90.6%) patients in the abatacept plus methotrexate group and 227 (89.7%) patients in the methotrexate group completed year 1 (fig 1). Fewer patients receiving abatacept plus methotrexate discontinued due to lack of efficacy (0% vs 3.2%) or adverse events (3.5% vs 4.3%), than those receiving methotrexate alone.

**Table 1:** Summary of baseline demographics, clinical characteristics and concomitant medications

	Abatacept + methotrexate	Placebo + methotrexate
	(n = 256)	(n = 253)
Age in years, mean (SD)	50.1 (12.4)	49.7 (13.0)
Gender, % female	76.6	78.7
Race, % white	78.9	86.6
Geographical region, n (%)		
North America, n (%)	46 (18.0)	40 (15.8)
South America, n (%)	103 (40.2)	102 (40.3)
Europe, n (%)	88 (34.4)	95 (37.5)
Rest of world, n (%)	19 (7.4)	16 (6.3)
Disease duration, mean months (SD)	6.2 (7.5)	6.7 (7.1)
Tender joints, mean (SD)	31.3 (14.8)	30.8 (14.0)
Swollen joints, mean (SD)	22.9 (11.3)	21.9 (10.1)
CRP levels, mg/dl, mean (SD)	3.1 (3.1)	3.6 (5.0)
DAS28 (CRP)*, mean (SD)	6.3 (1.0)	6.2 (1.0)
HAQ-DI (0–3)†, mean (SD)	1.7 (0.7)	1.7 (0.7)
RF positive, n (%)	246 (96.1)	245 (96.8)
Anti-CCP2 positive, n (%)	236 (92.2)	217 (85.8)
RF and anti-CCP2 positive, n (%)	227 (88.7)	211 (83.4)
Total *x* ray score‡, mean (SD) (maximum 290)	7.5 (9.7)	6.7 (8.8)
Total *x* ray score‡, median (range)	3.4 (0.0–57.3)	3.8 (0.0–54.6)
Erosion score‡, mean (SD) (maximum 145)	5.4 (6.1)	4.8 (5.4)
Erosion score‡, median (range)	2.9 (0–29.1)	3.1 (0–36.9)
Joint space narrowing‡, mean (SD) (maximum 145)	2.1 (4.2)	1.9 (4.0)
Joint space narrowing‡, median (range)	0.2 (0–28.2)	0.5 (0–28.0)
Total patients on antirheumatic concomitant medications at randomisation, n (%)	243 (94.9)	236 (93.3)
Corticosteroids, oral and/or injectable	131 (51.2)	124 (49.0)
Corticosteroids, oral <10 mg/day	95 (37.1)	86 (34.0)
NSAID	203 (79.3)	201 (79.4)
Other non-biological DMARD	7 (2.7)	10 (4.0)
Chloroquine	3 (1.2)	4 (1.6)
Hydroxychloroquine	4 (1.6)	5 (2.0)
Sulfasalazine	0	1 (0.4)

*n  =  252 for methotrexate; †n  =  254 for abatacept plus methotrexate, n  =  251 for methotrexate; ‡n  =  253 for abatacept plus methotrexate.

CCP2, cyclic citrullinated peptide type 2; CRP, C-reactive protein; DAS28, disease activity score in 28 joints; DMARD, disease-modifying antirheumatic drug; HAQ-DI, health assessment questionnaire disability index; NSAID, non-steroidal anti-inflammatory drug; RF, rheumatoid factor.

### Concomitant antirheumatic medications

Concomitant medications at baseline are shown in table 1. At month 6, the mean dose of methotrexate was 18.9 mg/week (SD 3.2) in the abatacept plus methotrexate group and 18.9 mg/week (SD 3.4) in the methotrexate group. At year 1 the mean dose of methotrexate was 18.1 mg/week (SD 4.2) in the abatacept plus methotrexate group and 19.0 mg/week (SD 2.3) in the methotrexate group. The addition of non-biological DMARD was permitted after month 6: six (2.3%) patients in the abatacept plus methotrexate group and 17 (6.7%) patients in the methotrexate group received additional non-biological DMARD. Over 1 year, 136 (53.1%) patients in the abatacept plus methotrexate group and 136 (53.8%) patients in the methotrexate group received oral steroids, and the mean dose was 6.7 mg (SD 3.2) versus 8.3 mg (SD 7.1), respectively. A total of 10 (3.9%) patients in the abatacept plus methotrexate group and 20 (7.9%) patients in the methotrexate group received oral steroid pulses (>10 mg); the mean dose of these was 18.2 mg (SD 4.7) versus 27.7 mg (SD 16.7), respectively.

### Efficacy

#### Clinical efficacy

**Figure 2:**
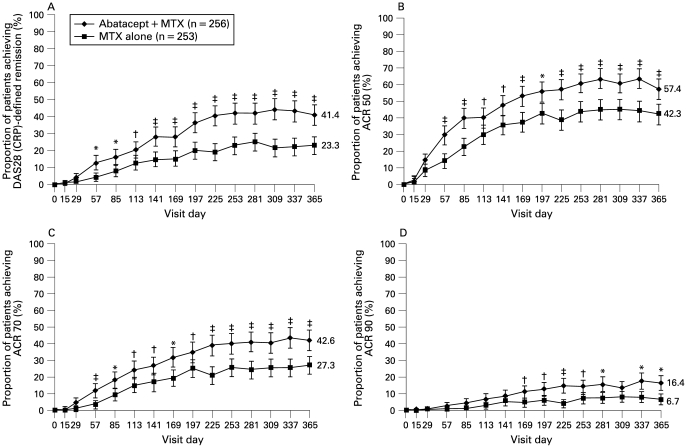
Summary of clinical efficacy. Proportion of patients in the abatacept plus methotrexate (MTX) and methotrexate alone groups over 1 year achieving (A) Disease activity score in 28 joints (DAS28, C-reactive protein)-defined remission; (B) American College of Rheumatology (ACR) 50; (C) ACR70; and (D) ACR90. Data are based on the intent-to-treat population, including all patients randomly assigned and treated. Patients who discontinued were considered non-responders. p Values represent abatacept plus methotrexate versus methotrexate alone. Error bars represent 95% CI. *p<0.01; †p<0.05; ‡p<0.001.

A significantly higher proportion of patients in the abatacept plus methotrexate group achieved DAS28 (CRP)-defined remission compared with the methotrexate group by day 57 (fig 2A), and a significant difference was maintained up to year 1 (41.4% vs 23.3%, respectively, at year 1; p<0.001). When patients for whom a non-biological DMARD was initiated were considered non-responders, the proportion of responders was 41.0% (95% CI 35.0 to 47.0) in the abatacept plus methotrexate group compared with 21.7% (95% CI 16.7 to 26.8) in the methotrexate group. At year 1, disease activity was significantly reduced with abatacept plus methotrexate compared with methotrexate alone (adjusted mean changes from baseline in DAS28 (CRP) were −3.22 (SE 0.09) and −2.49 (SE 0.09), respectively, p<0.001). The proportion of patients achieving ACR50 or ACR70 responses was significantly higher in the abatacept plus methotrexate compared with the methotrexate group by day 57 (fig 2B and C), and a significant difference was maintained up to year 1. For abatacept plus methotrexate compared with methotrexate alone at year 1, the proportion of patients achieving ACR50 was 57.4 versus 42.3% (p<0.001), ACR70 42.6 versus 27.3% (p<0.001) and ACR90 16.4 versus 6.7% (p = 0.001; fig 2B–D). Over 1 year, 27.3% of patients achieved an MCR versus 11.9% of patients receiving methotrexate alone (p<0.001).

#### Radiographic progression

**Figure 3:**
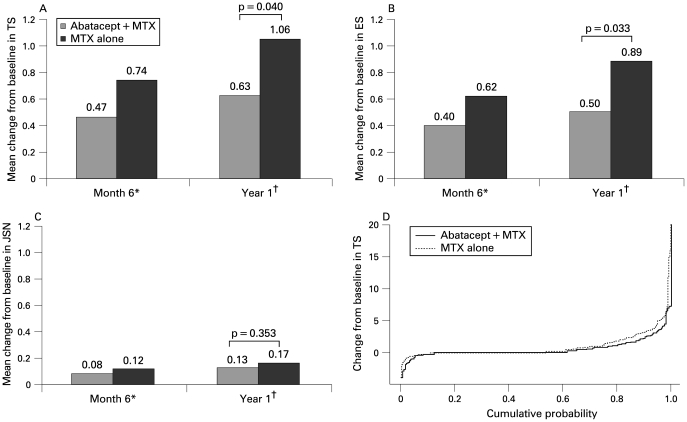
Inhibition of radiographic progression. Mean change from baseline at month 6 and year 1 for (A) total score (TS); (B) erosion score (ES); (C) joint-space narrowing (JSN) and (D) cumulative probability distribution of changes from baseline in Genant-modified total Sharp scores by treatment at year 1. p Values represent abatacept plus methotrexate (MTX) versus methotrexate alone at year 1. *Based on as-observed data. †Missing data were imputed by linear extrapolation.

Baseline radiographic scores are shown in table 1. At month 6 and year 1, observed data were available for 234 (91.4%) and 218 (85.2%) patients in the abatacept plus methotrexate group and 235 (92.9%) and 223 (88.1%) patients in the methotrexate group, respectively. Figure 3 presents mean changes in Genant-modified Sharp scores at month 6 (based on observed data) and year 1 (linear extrapolation applied). At year 1, changes from baseline in TS and ES were significantly lower in the abatacept plus methotrexate group compared with the methotrexate group (p = 0.040 and p = 0.033, respectively; fig 3A and B); mean changes in JSN scores were minimal and were comparable between the two groups (p = 0.353; fig 3C). Results were comparable when only completers were considered at the 1-year time point (mean changes from baseline in the abatacept plus methotrexate vs the methotrexate groups were 0.57 vs 1.05 (p = 0.020) for TS, 0.46 vs 0.89 (p = 0.025) for ES and 0.11 vs 0.16 (p = 0.246) for JSN, respectively). The median change from baseline in TS, ES and JSN was 0 for both groups. The cumulative probability plot (fig 3D) shows the distribution of change from baseline in TS over 1 year. At year 1, the proportion of patients with no radiographic progression (TS ⩽0) was 61.2% (95% CI 55.0 to 67.3) in the abatacept plus methotrexate group versus 52.9% (95% CI 46.6 to 59.2) in the methotrexate group, with an estimated difference of 8.3% (95% CI −1.0 to 17.5). When patients for whom a non-biological DMARD was initiated were considered progressors, the proportion of patients without progression was 59.1% (95% CI 52.9 to 65.3) compared with 48.3% (95% CI 42.1 to 54.6) for the abatacept plus methotrexate versus methotrexate groups, and the estimate of difference was 10.7% (95% CI 1.4 to 20.0). When patients who received steroid pulses were also considered progressors, the proportion of patients without progression was 58.3% (95% CI 52.1 to 64.5) compared with 47.1% (95% CI 40.8 to 53.4) and the estimate of difference was 11.2% (95% CI 1.8 to 20.5).

#### Physical function and HRQoL

At year 1, 184 (71.9%) patients in the abatacept plus methotrexate group compared with 157 (62.1%) patients in the methotrexate group achieved a change from baseline of 0.3 units or greater in HAQ-DI (p = 0.024 for the abatacept plus methotrexate versus methotrexate groups). The adjusted mean change from baseline in HAQ-DI was −0.96 (SE 0.04) for the abatacept plus methotrexate group and −0.76 (SE 0.04) for the methotrexate group, and the adjusted treatment difference did not cross zero (−0.20; 95% CI −0.31 to −0.08). Significant improvements in the SF-36 were also observed: the adjusted mean change from baseline was 11.68 (SE 0.62) versus 9.18 (SE 0.63) for the physical component summary and 8.15 (SE 0.64) versus 6.34 (SE 0.64) for the mental component summary (p = 0.005 for the physical component summary and p = 0.046 for the mental component summary for the abatacept plus methotrexate versus methotrexate groups).

### Safety

At year 1, the frequency of adverse events, serious adverse events and discontinuations as a result of serious adverse events was comparable between groups (table 2). The proportion of adverse events that led to discontinuation was 3.1% in the abatacept plus methotrexate group compared with 4.3% in the methotrexate group. The most frequently reported adverse events (>10% of patients in the abatacept plus methotrexate group) were nausea, upper respiratory tract infection and headache. There were six deaths; two (0.8%) in the abatacept plus methotrexate group and four (1.6%) in the methotrexate group. Of the two deaths in the abatacept plus methotrexate group, one patient had pneumonia and severe gastrointestinal bleeding and the other had an acute myocardial infarction.

**Table 2:** Summary of safety at year 1

	Abatacept + methotrexate (n = 256)	Placebo + methotrexate (n = 253)
	n (%)	n (%)
Adverse events	217 (84.8)	211 (83.4)
Discontinuations due to adverse events	8 (3.1)	11 (4.3)
Serious adverse events	20 (7.8)	20 (7.9)
Discontinuations due to serious adverse events	3 (1.2)	3 (1.2)
Infections	132 (51.6)	139 (54.9)
Serious infections	5 (2.0)	5 (2.0)
Autoimmune events	6 (2.3)	5 (2.0)
Acute infusional events	16 (6.3)	5 (2.0)
Malignancies	1 (0.4)	0 (0.0)
Deaths	2 (0.8)	4 (1.6)

The most frequent infections were upper respiratory tract infection in 26 (10.2%) versus 26 (10.3%) patients, nasopharyngitis in 21 (8.2%) versus 26 (10.3%) patients and influenza in 19 (7.4%) versus 23 (9.1%) patients in the abatacept plus methotrexate versus methotrexate groups, respectively. Serious infections were experienced by five (2.0%) patients in the abatacept plus methotrexate group (pneumonia, gastroenteritis, cellulitis, pseudomonal lung infection and postoperative wound infection, one patient each) and five (2.0%) patients in the methotrexate group (pneumonia, three patients; gastroenteritis, one patient; and breast cellulitis and staphylococcal infection, both in the same patient). No patients in the abatacept plus methotrexate group discontinued due to an infection. No opportunistic infections or cases of tuberculosis were reported in either group.

One malignancy was reported—pancreatic cancer in the abatacept plus methotrexate group. The patient was 67 years of age and had received 12 infusions of abatacept (750 mg) before the event. The event was considered unlikely to be related to study medication and abatacept was discontinued.

Six patients (2.3%) in the abatacept plus methotrexate group and five patients (2.0%) in the methotrexate group had adverse events that were recorded under the category of autoimmune disorders. Sjogren’s syndrome, sicca syndrome, systemic lupus erythematosus, psoriasis and atrophic gastritis occurred in one patient in each group; erythema nodosum occurred in one patient in the abatacept plus methotrexate group.

Sixteen patients (6.3%) in the abatacept plus methotrexate group experienced acute infusional events compared with five patients (2.0%) in the methotrexate group. The most common events were dizziness (five (2.0%) vs two (0.8%) patients for abatacept plus methotrexate vs methotrexate alone, respectively); all other events were reported in two or less than two patients in either group. All acute infusional events were mild or moderate in intensity, except for one case of severe urticaria in the abatacept plus methotrexate group.

Two abatacept patients became pregnant (protocol violations). One patient had received one abatacept infusion, had a positive urine pregnancy test on day 1 and experienced a spontaneous abortion between days 1 and 30. One patient received nine infusions of abatacept and had a positive urine pregnancy test on day 253. Pregnancy was confirmed by ultrasound and was subsequently terminated by induced abortion on day 281. Both patients were discontinued from the study as a result of pregnancy.

## Discussion

This is the first trial to examine the impact of the modulation of T-cell co-stimulation with abatacept in patients with early RA. Both primary endpoints were met. Abatacept plus methotrexate was significantly better at inducing DAS28 (CRP)-defined remission and reducing radiographic progression compared with methotrexate alone. Sensitivity analyses demonstrated that background medications did not impact the primary endpoints. Consistent with previous studies of abatacept in patients with established RA,^22^ ^23^ clinical responses (ACR criteria) and clinically meaningful improvements in both physical function and HRQoL were significantly higher in the abatacept plus methotrexate group compared with the group receiving methotrexate alone. In addition, the safety and tolerability of abatacept plus methotrexate was generally comparable to methotrexate alone.

The clinical benefits observed with abatacept plus methotrexate were coupled with significantly lower rates of radiographic progression compared with the methotrexate-alone group. In a patient population with established RA and an inadequate response to methotrexate from the Abatacept in Inadequate Responders to Methotrexate (AIM) study, the rate of structural damage progression was significantly lower during the second year of treatment compared with the first year.^19^ The 2-year results from the current trial will determine whether this effect is also observed in patients with early RA. In contrast to the lower rates of progression in TS and ES in the abatacept plus methotrexate group, rates of JSN progression were minimal in both groups and were not significantly different. Consistent with other trials in patients with early RA,^6^ ^7^ ^8^ ^9^ baseline values and the progression of JSN scores were low in both treatment arms, and it is possible that there was not enough power to detect small differences in rates of progression between the two groups.

On-drug remission is now an important and realistic therapeutic goal, which is increasingly reflected in the design of clinical trials for RA. This abatacept study and an early RA etanercept trial^10^ are the first studies of biological agents to use DAS28-defined remission as a primary endpoint. Remission was also the primary endpoint of an early RA trial comparing combination non-biological DMARD therapy with monotherapy,^24^ although in that trial remission was defined using the ACR criteria.^25^ The clinical and radiographic efficacy benefits reported here, including the proportion of patients achieving DAS28 (CRP)-defined remission, are comparable with those observed in other similar trials of early RA with biological therapies.^6^ ^7^ ^8^ ^10^ This is particularly encouraging because of the poor prognostic status of the patients in this study, as all were required to have erosions and to be RF or anti-CCP2-seropositive at baseline. In fact, approximately 90% of the patients were anti-CCP2 positive. These prognostic factors all correlate with poor long-term outcomes and an aggressive disease course.^2^ ^3^

As a result of the chronic long-term nature of RA, the safety and tolerability of a therapeutic agent is of critical importance. In this study, the overall frequency of adverse events and serious adverse events (including serious infections and autoimmune events) was similar between treatment groups, and in some cases was numerically lower in the abatacept plus methotrexate group. Infusional reactions occurred more frequently in the abatacept plus methotrexate group and were mostly mild in severity. Across both groups there were no cases of tuberculosis or opportunistic infections. One case of cancer was reported across treatment arms (pancreatic cancer in the abatacept plus methotrexate group). These data are consistent with the safety findings in previous studies of abatacept-treated patients with RA of a longer disease duration.^22^ ^23^ The safety and tolerability of abatacept plus methotrexate in this study was further supported by a high retention rate (>90%) and a low rate of discontinuations for safety reasons.

These data should be interpreted within the context of the trial. As these short-term, double-blind data are restricted to 1 year, the capacity to assess longer-term structural changes or detect infrequent safety-related events may be limited. It will be interesting to examine this group of poor prognosis patients over the second year of this 2-year trial in order to monitor whether the clinical, functional and radiographic benefits observed here are maintained or improved, as has been observed with abatacept in patients with established RA.^13^ ^14^ As this study focused on a poor prognosis patient population who were seropositive for RF and/or anti-CCP, seronegative patients were not included. However, it should be noted that abatacept has previously been demonstrated to have efficacy in patients with established RA in a number of trials that have included both seropositive and seronegative patients.^13^ ^14^

In summary, the combination of abatacept plus methotrexate was significantly more effective in inducing DAS28 (CRP)-defined remission and inhibiting radiographic progression than methotrexate alone in patients with early RA and poor prognostic factors. Coupled with a safety and tolerability profile comparable to methotrexate alone, these data support the early use of abatacept in RA patients with moderate to severe disease.

## References

[1] Kroot EJ de Jong BA van Leeuwen MA The prognostic value of anti-cyclic citrullinated peptide antibody in patients with recent-onset rheumatoid arthritis. Arthritis Rheum 2000;43:1831–5 10943873 10.1002/1529-0131(200008)43:8<1831::AID-ANR19>3.0.CO;2-6

[2] Meyer O Labarre C Dougados M Anticitrullinated protein/peptide antibody assays in early rheumatoid arthritis for predicting five year radiographic damage. Ann Rheum Dis 2003;62:120–6 12525380 10.1136/ard.62.2.120 PMC1754441

[3] Vittecoq O Pouplin S Krzanowska K Rheumatoid factor is the strongest predictor of radiological progression of rheumatoid arthritis in a three-year prospective study in community-recruited patients. Rheumatology (Oxford) 2003;42:939–46 12730503 10.1093/rheumatology/keg257

[4] Goekoop-Ruiterman YP de Vries-Bouwstra JK Allaart CF Clinical and radiographic outcomes of four different treatment strategies in patients with early rheumatoid arthritis (the BeSt study): a randomized, controlled trial. Arthritis Rheum 2005;52:3381–90 16258899 10.1002/art.21405

[5] Grigor C Capell H Stirling A Effect of a treatment strategy of tight control for rheumatoid arthritis (the TICORA study): a single-blind randomised controlled trial. Lancet 2004;364:263–9 15262104 10.1016/S0140-6736(04)16676-2

[6] St Clair EW van der Heijde DM Smolen JS Combination of infliximab and methotrexate therapy for early rheumatoid arthritis: a randomized, controlled trial. Arthritis Rheum 2004;50:3432–43 15529377 10.1002/art.20568

[7] Breedveld FC Weisman MH Kavanaugh AF The PREMIER study: a multicenter, randomized, double-blind clinical trial of combination therapy with adalimumab plus methotrexate versus methotrexate alone or adalimumab alone in patients with early, aggressive rheumatoid arthritis who had not had previous methotrexate treatment. Arthritis Rheum 2006;54:26–37 16385520 10.1002/art.21519

[8] Bathon JM Martin RW Fleischmann RM A comparison of etanercept and methotrexate in patients with early rheumatoid arthritis. N Engl J Med 2000;343:1586–93 11096165 10.1056/NEJM200011303432201

[9] Genovese MC Bathon JM Martin RW Etanercept versus methotrexate in patients with early rheumatoid arthritis: two-year radiographic and clinical outcomes. Arthritis Rheum 2002;46:1443–50 12115173 10.1002/art.10308

[10] Emery P Breedveld FC Hall S Comparison of methotrexate monotherapy with a combination of methotrexate and etanercept in active, early, moderate to severe rheumatoid arthritis (COMET): a randomised, double-blind, parallel treatment trial. Lancet 2008;372:375–82 18635256 10.1016/S0140-6736(08)61000-4

[11] Weisman MH Durez P Hallegua D Reduction of inflammatory biomarker response by abatacept in treatment of rheumatoid arthritis. J Rheumatol 2006;33:2162–6 17014006

[12] Gregersen PK Silver J Winchester RJ Genetic susceptibility to rheumatoid arthritis and human leukocyte antigen class II polymorphism. The role of shared conformational determinants. Am J Med 1988;85:17–19 3059797 10.1016/0002-9343(88)90374-9

[13] Kremer JM Genant HK Moreland LW Results of a two-year followup study of patients with rheumatoid arthritis who received a combination of abatacept and methotrexate. Arthritis Rheum 2008;58:953–63 18383390 10.1002/art.23397

[14] Genovese MC Schiff M Luggen M Efficacy and safety of the selective co-stimulation modulator abatacept following 2 years of treatment in patients with rheumatoid arthritis and an inadequate response to anti-tumour necrosis factor therapy. Ann Rheum Dis 2008;67:547–54 17921185 10.1136/ard.2007.074773

[15] Arnett FC Edworthy SM Bloch DA The American Rheumatism Association 1987 revised criteria for the classification of rheumatoid arthritis. Arthritis Rheum 1988;31:315–24 3358796 10.1002/art.1780310302

[16] Wells G Becker J-C Teng J Validation of the Disease Activity Score 28 (DAS28) and EULAR response criteria based on CRP against disease progression in patients with rheumatoid arthritis, and comparison with the DAS28 based on ESR. Ann Rheum Dis 2009;68:954–60 18490431 10.1136/ard.2007.084459 PMC2674547

[17] Genant HK Methods of assessing radiographic change in rheumatoid arthritis. Am J Med 1983;75:35–47 6660239 10.1016/0002-9343(83)90473-4

[18] Genant HK Jiang Y Peterfy C Assessment of rheumatoid arthritis using a modified scoring method on digitized and original radiographs. Arthritis Rheum 1998;41:1583–90 9751090 10.1002/1529-0131(199809)41:9<1583::AID-ART8>3.0.CO;2-H

[19] Genant HK Peterfy CG Westhovens R Abatacept inhibits progression of structural damage in rheumatoid arthritis: results from the long-term extension of the AIM trial. Ann Rheum Dis 2008;67:1084–9 18086727 10.1136/ard.2007.085084 PMC2569144

[20] Wells GA Tugwell P Kraag GR Minimum important difference between patients with rheumatoid arthritis: the patient’s perspective. J Rheumatol 1993;20:557–60 8478873

[21] Wells G Li T Maxwell L Determining the minimal clinically important differences in activity, fatigue, and sleep quality in patients with rheumatoid arthritis. J Rheumatol 2007;34:280–9 17304654

[22] Genovese MC Becker JC Schiff M Abatacept for rheumatoid arthritis refractory to tumor necrosis factor alpha inhibition. N Engl J Med 2005;353:1114–23 16162882 10.1056/NEJMoa050524

[23] Kremer JM Genant HK Moreland LW Effects of abatacept in patients with methotrexate-resistant active rheumatoid arthritis: a randomized trial. Ann Intern Med 2006;144:865–76 16785475 10.7326/0003-4819-144-12-200606200-00003

[24] Mottonen T Hannonen P Leirisalo-Repo M Comparison of combination therapy with single-drug therapy in early rheumatoid arthritis: a randomised trial. FIN-RACo trial group. Lancet 1999;353:1568–73 10334255 10.1016/s0140-6736(98)08513-4

[25] Pinals RS Masi AT Larsen RA Preliminary criteria for clinical remission in rheumatoid arthritis. Arthritis Rheum 1981;24:1308–15 7306232 10.1002/art.1780241012

